# The need for robust characterization of nanomaterials for nanomedicine applications

**DOI:** 10.1038/s41467-021-25584-6

**Published:** 2021-09-02

**Authors:** Morteza Mahmoudi

**Affiliations:** Department of Radiology and Precision Health Program, Michigan State University, East Lansing, MI USA

**Keywords:** Nanomedicine, Characterization and analytical techniques

## Abstract

Robust and precise characterization of the interactions between nanoengineered materials and biosystems is vital for the development of safe, efficient diagnostic and therapeutic nanomedicines. This comment discusses the key aspects of nanoparticle characteristics affecting the interpretation of nano-bio interface data.

## Intended use dictates adequate and accurate characterization of nanoparticles

**Fig. 1:**
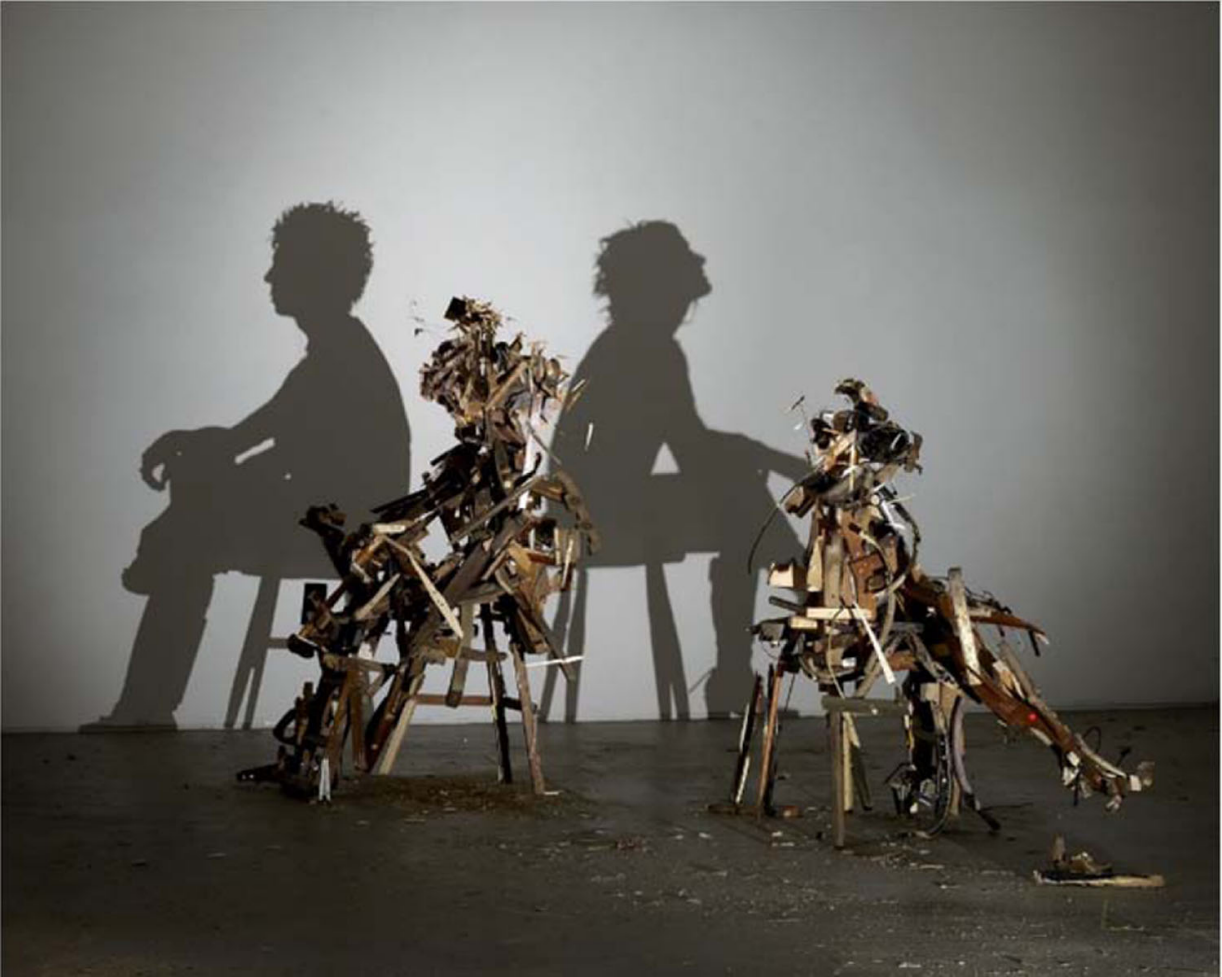
Example of how outcomes (in this case the projected shadows) of an incompletely characterized object may cause misinterpretation. Projected shadows of self-portraits created by ordinary things (e.g., rubbish). Tim Noble and Sue Webster, Wild Mood Swings, 2009–10; © Tim Noble and Sue Webster. All Rights Reserved, DACS/Artimage 2021. Photo: Andy Keate.

Incomplete characterization of an object may cause significant misinterpretation of outcomes (Fig. 1). Collecting accurate and valid information on the characterization of nanoparticles using the correct methodologies, is a key step towards a precise understanding and/or prediction of their safety and therapeutic/diagnostic efficacies. The type and extent of the characterization techniques, however, depend strongly on the intended use of the nanoparticles^1^, which is of crucial importance. For example, the nanotechnology regulatory science research plan guidance documents of the US Food and Drug Administration (FDA) state, “We intend our regulatory approach to be adaptive and flexible and to take into consideration the specific characteristics and the effects of nanomaterials in the *particular biological context* of *each product* and its *intended use*” (https://www.fda.gov/science-research/nanotechnology-programs-fda/fdas-approach-regulation-nanotechnology-products). Without using the correct characterization techniques that match the intended use, the outcomes may be misleading and lead to incorrect assumptions about the safety, blood residency, and therapeutic efficacy of nanoparticles.

For example, conventional characterization techniques (e.g., electron microscopies, dynamic light scattering, and zeta-potential) would identify patchy charged and uniform charged mesoporous silica nanoparticles as identical but can have significantly different in vivo outcomes. Patchy charged nanoparticles are reported to be immediately captured by white blood cells, while uniformly charged nanoparticles remain in circulation^2^.

## Failure to characterize purchased and common materials

Failure to robustly characterize the purchased, gifted, synthesized (using well-defined protocols), and/or common materials can cause significant errors in the results of the intended use of nanoparticles. This is mainly because the provided physical and chemical properties of the (nano)materials (e.g., by commercial supplier) may change significantly due to the (i) environmental and methodological factors (e.g., storage time and the employed characterization technique) and/or (ii) the essential buffering conditions for the intended use of nanoparticles (e.g., for protein corona preparation and analysis). For example, a commercial amine-modified polystyrene nanoparticle with the size of 100 nm (according to the manufacturer) demonstrated poor dispersity, and therefore much larger sizes, by dynamic light scattering (e.g., 4323 nm and 3293 nm in water and phosphate buffered saline, respectively) when recharacterized in laboratory;^3^ in addition, the ζ potential measurement of these positively charged nanoparticles in phosphate buffered saline demonstrated negative surface charge^3^. If these nanoparticles were used without recharacterization in the laboratory for protein corona preparation and analysis, the proteomics outcomes of such study would have been full of errors! To avoid errors and misinterpretation of the nanomedicine characteristics, accurate recharacterization of the purchased, gifted, and/or common materials in a compatible media for the intended use of nanoparticles is essential.

Aside from the need for accurate recharacterization of the purchased, gifted, and/or common nanoparticles, accurate and validated information on the biological systems (e.g., biological fluids and cells) is essential to achieve robust nanobio interfaces. This is mainly because the characteristics of biological systems can significantly affect the safety and diagnostic/therapeutic efficacy of nanomedicine. For example the biological interactions and the fate of the nanoparticles can be influenced by if the source is animal or human, if testing is done in serum, plasma or whole blood, if biological tissues and fluids are from healthy or diseased donors and even by if the samples are fresh or stored^4^. Rigorous authentication and reporting full information of the biological parameters are, therefore, another essential factor that can significantly reduce the heterogeneity surrounding nanomedicine reproducibility^1^.

## Characterization of nanobio interfaces

Obtaining robust data on nanoparticles’ size, charge, and other physical and chemical properties (e.g., colloidal stability and dissolution) before and after interactions with biological systems is simultaneously of particular interest and a substantial challenge. This is mainly because the biomolecular/protein corona formed on the surface of nanoparticles changes their physical and chemical properties and, therefore, determines their interactions with biological systems (e.g., immune system and cells)^5^. For example, formation of protein corona on the surface of silica nanoparticles weakens their adhesion to cell membrane and, thus, reduce nanoparticles’ uptake by the cells, as compared to the pristine nanoparticles^6^. Therefore, reports on a particular nanomedicine should include accurate data on both pristine and corona-coated nanoparticles, and the comprehensiveness of the presented data should depend on their intended use. In addition, because the nature of the protein corona evolves over time, continued characterization based on intended use may be essential. For example, for cancer therapeutic nanoparticles, evolution of the protein corona in the tumor microenvironment is of crucial interest in better understanding the interactions between nanoparticles and the immune system (e.g., macrophages^7^) and cancer cells.

For biomedical application, it is essential to conduct and report deep and combined analysis of the physical and chemical properties of nanoparticles, the biological parameters tested, and the nanobio interfaces (e.g., purity of protein corona). Without such comprehensive datasets, the safety and therapeutic efficacy nanoparticles cannot be accurately predicted and, therefore, are incompatible with regulatory guidelines.

It is substantially more difficult to perform robust characterization analysis of corona-coated nanoparticles than pristine nanoparticles, and the degree of difficulty depends on the characterization technique and factors affecting the results (Table 1). For example, analysis of the size of corona-coated nanoparticles using differential centrifugal sedimentation (DCS) (https://www.intertek.com/analysis-differential-centrifugal-sedimentation/), is more challenging than the pristine nanoparticles mainly because DCS requires that nanoparticles have a particular density. Although the density of some particles is well defined in their pristine state, the density of corona-coated nanoparticles cannot be defined accurately due to the significant debate/challenges regarding the density of proteins^8–11^ within the corona layer. As another example, common microscopy images often fail to define (protein) coating boundaries, yielding underestimated sizes; while light scattering can produce overestimates^1^. Therefore, reporting both datasets on pristine and corona-coated nanoparticles (with proper experimental and analytical replications) will improve our understanding of the characteristics of the nan-bio interface (e.g., their chemical and physical properties, formation of aggregates, and protein impurities), providing better clarity regarding the biological responses to nanoparticles^1^. While even subtle changes (e.g., functionalization groups) may not have a significant effect on the characteristics of pristine nanoparticles, it can result in a completely different protein corona composition^12^ and substantially alter the safety and therapeutic/diagnostic efficacy of nanoparticles.

**Table 1:** List of the common analytical methods used to study the nanobio interface and related factors that can significantly affect their outcomes (details of each method can be found in refs. ^14,24^).

Analytical Methods	Factors that significantly affecting analytical results
Atomic force microscope	Probe and operating mode that are not matched with nanoparticles physical and chemical properties; vibration; various sources of artifacts (e.g., tip and scanner); image processing software; parameter setting.
Chromatography	Improper setup and/or contamination (even at the nanogram scale)
Circular dichroism	Impurity of the single protein solution
Differential centrifugal sedimentation	Density measurement of the corona-coated nanoparticles Nanoparticle shape
Dynamic light scattering	Hydration shells Nanoparticle shape Counterion binding Size dispersity of nanoparticles
Electrophoresis	Inner surface characteristics of the capillary (which affect protein adsorption)
Fluorescence spectroscopy	Stability of the fluorescence tag
Fourier transform infrared Spectroscopy	Complexity of the media Sample preparation
Isothermal titration calorimetry	Sample concentration
Magnetic levitation	Paramagnetic solution Concentration of (super)paramagnetic ions/nanoparticles
Mass spectrometry	Expertise and experience of the operator
Nuclear magnetic resonance spectroscopy	Expertise and experience in interpretation of the outcomes
Quartz crystal microbalance	Variations in interfacial parameters (e.g., surface roughness, surface free energy, surface charge, and viscoelasticity)
Raman spectroscopy	Fluorescence of impurities or of the sample Sample heating through laser radiation
Surface plasmon resonance	Size and affinity of biomolecules
UV/vis	Solution features (e.g., composition, pH, electrolyte concentrations, and impurities) environmental (e.g., temperature) parameters Experimental variations [e.g., slit width (effective bandwidth) of the spectrophotometer] will also alter the spectrum
ζ potential	Ionic strength Size dispersity of nanoparticles

To overcome issues associated with any single technique, researchers should use at least one other complementary characterization technique to overcome the weaknesses of a single technique. For example, DCS analysis could be paired with a complementary characteristics approach such as cryo-transmission electron microscopy^13^ for more accurate analysis of the thickness of the protein corona.

Another critical aspect is to collaborate/consult with experts in each characterization technique; results that seem dramatic to non-experts may be only artifacts to expert eyes! For example, characterization images of nanoparticles using atomic force microscopy (AFM) technique may contain significant series of artifacts (arisen from tip (e.g., repetitive abnormal patterns in an image), scanner (e.g., height measurements), vibration, feedback and parameter settings, and image processing software)^14^ that may not be identified by non-AFM experts (who may be experts in other conventional characterization techniques). An AFM experts, on the other hand, can easily recognize the source of the artifact provide testing guidelines (e.g., by taking more images and changing scan direction, size, and speed)^14^ to produce artifact-free images.

Another important aspect of characterization is the role of interlaboratory variations. It has been shown that the characteristics outcomes of identical nanoparticles obtained by different laboratories were significantly different^15^. To avoid possible confusion caused by interlaboratory variations, one strategy is to recheck the characterization outcomes with standard labs including National Cancer Institute’s Nanotechnology Characterization Lab (NCI-NCL; https://ncl.cancer.gov/).

## Standardization of nanoparticles’ characterization

Standardization of characterization techniques (e.g., report checklist) is being considered in the field of nanomedicine, in order to ensure the accuracy, reliability, and reproducibility of the outcomes presented and also to enable meta-analysis evaluation of the literature and comparison of experimental data^4^. For example, Caruso and co-workers^16^ proposed the checklist referred to as Minimum Information Reporting in Bio-Nano Experimental Literature (MIRIBEL) to improve the reproducibility and robustness of nanomedicine reports. Various responses from experts in the field^1^, despite supporting the initial goal of the MIRIBEL checklist, demonstrated the need for a list more closely tailored to the intended use of nanoparticles. Such tailored checklists can be developed only by integrated functioning of all stakeholders (e.g., scientists, funding agencies, and regulatory organizations), with timely and effective creation of optimal protocols to maximize reproducibility and transparency in nanomedicine. Until stakeholders agree on tailored checklists for specific nanoparticles and their particular applications, the best strategy is to follow the following proposed guidelines for transparency, openness, and reproducibility, which addresses eight standards: citations, data transparency, analytic methods (code) transparency, research materials transparency, design and analysis transparency, preregistration of studies, preregistration of analysis plans, and replication^17^.

It is noteworthy, however, that providing robust and comprehensive characterization data (e.g., conducting at least two different techniques for each characteristic feature and performing the same test in different laboratories) may be challenging for early-career scientists, as it requires critical resources more likely to be available to senior scientists/faculties, e.g., the amount of funding, collaborative network, their interdisciplinary capacity, and long-term experience in using various characteristics techniques^18^. Therefore, the scientific community needs to ensure that such a tailored standardization process would not hinder research breakthroughs from scientists/faculty members who may not yet have ready access to all such resources^18^.

## Translational industry reports require comprehensive and robust characterization data

Although reports on nanomedicines (and in general, all scientific fields) must have the highest possible standard based on their intended use, more strict attention and emphasis should be placed on translational industry reports. This is mainly because of the direct influence of these reports on healthcare systems and the needs of regulatory organizations to access all relevant data to better assess the safety and efficacy of translational products. As stated by FDA’s nanotechnology regulatory guidelines:

“Industry remains responsible for ensuring that its products meet all applicable legal requirements, including safety standards. … industry must work with current information in product development, and continue to monitor products once marketed.” (https://www.fda.gov/science-research/nanotechnology-programs-fda/fdas-approach-regulation-nanotechnology-products)

There are considerable transparency and reproducibility issues regarding the current literature in nanomedicine^1,16^. This is, at least in part, due to the fact that published articles contain “polished narratives which convey the impression that everything went smoothly according to the proposed research plan”^19^, which increases the risk of having considerable wrong or false conclusions. This issue in translational industry reports is more concerning than the current literature, mainly because of the possibility of financial conflicts of interest and serious consequences to the healthcare system and patients’ health. It is noteworthy that reproducibility problems in clinical trials are not limited to nanomedicine; for example, the rate of reproducibility among 67 preclinical studies in general biology was reported to be only 20–25%^20^. As another example, reproducibility checks of cancer biology projects revealed that, among the five most highly cited research studies in the field, only two could be successfully reproduced^21,22^. Clear and transparent discussion about reports helps regulatory decision-makers consider all aspects of the reports in making decisions that may affect healthcare. Such discussions require integrated functioning among all stakeholders including the scientific community (e.g., reviewers, editors, and readers) to identify insufficiently comprehensive and/or misleading science through the peer-review and post-publication processes.

In summary, the central message of this comment paper is to shed more light on the reproducibility, robustness, and comprehensiveness/integrity of nanomedicine characterization reports, which may cause serious misinterpretation in prediction of nanoparticles’ safety and diagnostic/therapeutic efficacy. Achieving reproducibility and transparency during the characteristics of nanomedicine products may improve the current low rate of success in clinical translation of nanomedicine products^23^. To begin to address these issues in a timely and effective manner, I call upon stakeholders (e.g., researchers, reviewers, editors, institutions, funding agencies, entrepreneurs, and media) to work together to improve transparency, openness, and reproducibility across the field of nanomedicine.
